# Antinutritional factors, nutritional improvement, and future food use of common beans: A perspective

**DOI:** 10.3389/fpls.2022.992169

**Published:** 2022-08-23

**Authors:** Eleonora Cominelli, Francesca Sparvoli, Silvia Lisciani, Chiara Forti, Emanuela Camilli, Marika Ferrari, Cinzia Le Donne, Stefania Marconi, Barend Juan Vorster, Anna-Maria Botha, Diana Marais, Alessia Losa, Tea Sala, Emmanuelle Reboul, Katherine Alvarado-Ramos, Boaz Waswa, Beatrice Ekesa, Francisco Aragão, Karl Kunert

**Affiliations:** ^1^National Research Council, Institute of Agricultural Biology and Biotechnology, Milan, Italy; ^2^Council for Agricultural Research and Economics, Research Centre for Food and Nutrition, Rome, Italy; ^3^Department Plant and Soil Sciences, Forestry and Agricultural Biotechnology Institute, University of Pretoria, Pretoria, South Africa; ^4^Department of Genetics, Stellenbosch University, Stellenbosch, South Africa; ^5^Council for Research in Agriculture and Economics, Research Centre for Genomics and Bioinformatics, Montanaso Lombardo, Italy; ^6^Aix-Marseille University, INRAE, INSERM, C2VN, Marseille, France; ^7^International Center for Tropical Agriculture (CIAT), CIAT Regional Office for Africa, Nairobi, Kenya; ^8^Embrapa Recursos Genéticos e Biotecnologia, Brasília, Brazil

**Keywords:** common bean, nutritional quality, bioactive compounds, antinutrients, mutants, phytic acid, lectins

## Abstract

Common bean seeds are an excellent source of protein as well as of carbohydrates, minerals, vitamins, and bioactive compounds reducing, when in the diet, the risks of diseases. The presence of bioactive compounds with antinutritional properties (e.g., phytic acid, lectins, raffinosaccharides, protease inhibitors) limits, however, the bean’s nutritional value and its wider use in food preparations. In the last decades, concerted efforts have been, therefore, made to develop new common bean genotypes with reduced antinutritional compounds by exploiting the natural genetic variability of common bean and also applying induced mutagenesis. However, possible negative, or positive, pleiotropic effects due to these modifications, in terms of plant performance in response to stresses or in the resulting technological properties of the developed mutant genotypes, have yet not been thoroughly investigated. The purpose of the perspective paper is to first highlight the current advances, which have been already made in mutant bean characterization. A view will be further provided on future research directions to specifically explore further advantages and disadvantages of these bean mutants, their potential use in innovative foods and representing a valuable genetic reservoir of combinations to assess the true functional role of specific seed bioactive components directly in the food matrix.

## Introduction

Today’s consumers’ dietary patterns are no longer sustainable, which has a strong impact on both planetary and human health. Animal-derived proteins further account for almost 40% of humanity’s total protein consumption. This consumption is expected to dramatically increase as a result of the world population rise (Henchion et al., 2017). Concerns about the environmental costs of livestock farming and the effects of consuming such a large amount of meat on human health (Godfray et al., 2018) are refocusing political, societal and scientific attention with the aim to search for alternative protein sources. Pulses are, in this regard, excellent candidates for this transition. They are both sustainable and healthy food crops (Foyer and Noctor, 2016; Bessada et al., 2019) and, with FAO (Food and Agriculture Organization of the United Nations) assistance, about 100 countries have already developed food-based dietary guidelines.^1^ However, very few mention pulses, as an important source of protein that could greatly reduce future meat consumption (Rawal and Navarro, 2019). Despite this obvious benefit, consumption of pulses has precipitously declined in recent decades, especially in developed countries (Messina, 2014). Consumers are particularly hesitant to eat pulses due to their concern that pulses contain antinutrients, cause flatulence, have long cooking times (Doma et al., 2019). But also the lack of knowledge of how to prepare pulses as a meal as well as the perception that pulses are “poor person’s food” has contributed to this hesitance (Cichy et al., 2019; Doma et al., 2019). During the COVID-19 pandemic, demand for pulses interestingly increased (Di Renzo et al., 2020; Lucier and Parr, 2020; Ruiz-Roso et al., 2020) which very likely indicates that consumers are indeed aware of the benefits of pulses as being shelf-stable, economical, and healthy. Offering improved quality pulses and innovative food preparations containing legumes can, therefore, be an attractive way to fuel consumer motivation and ensure that this positive trend of pulse consumption continues (Didinger and Thompson, 2020).

Pulses and common bean (*Phaseolus vulgaris* L.) in particular, play a key role in the traditional diets, in less developed countries of the world (Murube et al., 2021; Singh et al., 2021). Common beans are high in protein and low in fats, sodium and calories (Celmeli et al., 2018; Kibar and Kibar, 2019). Additionally, the presence of group B vitamins, the consistent amounts of minerals and trace elements (Hayat et al., 2014; Kouris-Blazos and Belski, 2016; Celmeli et al., 2018), the high quantities of starch and fiber, as well as of specific protein fractions responsible for important physiological and metabolic effects (Fujiwara et al., 2017) is a significant additional value of common beans. Finally, numerous bioactive compounds in beans, such as polyphenols, flavonoids, anthocyanins and carotenoids, have been related to prevention and/or regulation of chronic diseases when beans are consumed on a regular basis (Golam Masum Akond et al., 2011; Messina, 2014; Kibar and Kibar, 2019; Mullins and Arjmandi, 2021). However, the presence of different so-called antinutritional factors (ANFs), which are non-nutrients or bioactive compounds greatly limit the nutritional value of common bean seeds (Campos-Vega et al., 2011; Carbas et al., 2020). Common bean ANFs include: phytate among the major constrain to mineral cation bioavailability; polyphenols, interfering with nutrient absorption; lectins, raffinose family oligosaccharides and saponins, causing gastrointestinal discomfort with lectins resulting toxic if beans are not properly cooked; bioactive peptides and enzyme inhibitors interfering with protein digestibility and bioavailability, respectively (Wiesinger et al., 2022).

Aim of our Perspective paper is to first highlight the advances that have been so far made to lower, or eliminate, these ANFs from common bean seeds. As these compounds have also specific functions in the plant by, for example, protecting against biotic and abiotic stresses, we will highlight such aspects which should, in our view, not be underestimated in any bean breeding program. We will then discuss how the population target and the specific food formulations will benefit from the use of ANF-changed beans and how these beans can be used as an alternative to animal proteins and possibly partly replacing in the longer-term these proteins. Finally, we will also present our view on future research directions to specifically explore additional advantages and disadvantages of common bean mutant lines low in ANFs and discussing the possible potential uses of these lines in innovative foods.

## Use of common bean

Common beans are traditionally used in the preparation of soups, salads and are sometimes also combined with cereals. Few examples are further available concerning the application of common bean in alternative food preparations. The use of beans in the preparation of ready-to-eat bakery food products, meat-type derived products (Mecha et al., 2021) as well as in some country traditional recipes normally prepared with other ingredients (e.g., pasta for Italy) could, however, promote worldwide the increment of bean protein consumption. This can happen by not only considering the beans’ nutritional but also its functional properties, such as solubility, water/oil holding capacity, viscosity, and mainly the excellent emulsifying properties of bean proteins (Foschia et al., 2017; Rahmati et al., 2018).

## Improvement for antinutritional factors content

Although a number of bean products have been already developed (Giuberti et al., 2015; Pérez-Ramírez et al., 2018; Mecha et al., 2021), by-products, such as bean flour, will likely contain ANFs requiring an appropriate processing technology to eliminate them (Rudraraju et al., 2021). A further major obstacle to directly use common bean flour without any processing is the presence of lectins. Lectins, which are encoded by a small multigene family (APA locus; Arcelin/Phytohemagglutinin/alpha-Amylase inhibitor), consist of true lectins (PHA-E and PHA-L) and also proteins, α-amylase inhibitor (αAI) and arcelins (Arc), which are structurally similar but with different properties (Lioi et al., 2003). α-AI is traditionally considered as an antinutrient limiting starch assimilation in livestock feeding. Inhibitor activity is, however, exploited for the production of starch blockers to control body weight gain (Peddio et al., 2022). In contrast, arcelins are only present in some wild bean genotypes and may confer seed resistance against the attack by phytophagous insects (Zaugg et al., 2013). All APA proteins are further highly resistant to enzymatic proteolysis (Marquez and Lajolo, 1981; Jivotovskaya et al., 1996). Heat treatment improves, however, their hydrolysis, but a residual activity causing reduced protein digestibility and toxicity can also sometimes be detected after cooking (Bender and Reaidi, 1982; Petry et al., 2016; Sparvoli et al., 2016).

Bean breeding lines devoid of active PHA (“lectin null”, *lec*^–^) were obtained by introgression of an APA locus carrying an inactive PHA, the so called “pinto lectin,” and an active αAI (Confalonieri et al., 1992). Improved protein digestibility and a protein digestibility corrected amino acid score of *lec*^–^ mutant bean lines has been found after feeding rats with raw and cooked beans derived from *lec*^–^ genotypes (Bollini et al., 1999). In addition, the availability of the *lec*^–^ common bean flour has opened new possibilities for the direct use of an innovative flour in baked product preparations avoiding any previous flour processing (Sparvoli et al., 2016, 2021). Snacks obtained with common bean flour from these genotypes are also more protein rich than snacks from traditional flour (Sparvoli et al., 2016, 2021). However, baking not fully inactivates α-AI that very likely contributes to lowering the predicted biscuit glycemic index (Sparvoli et al., 2016, 2021). Therefore, genetic elimination of lectins, while keeping an active α-AI, has, in our view, a great potential to promote the use of common beans also in food preparations in which it is not traditionally used.

Furthermore, the isolation of a *low phytic acid* (*lpa1*) mutant with up to 90% less phytic acid (PA), a strong mineral cation chelator, has been another important step in improving the nutritional quality of common beans (Campion et al., 2009, 2013; Petry et al., 2013). A bean line combining the *lec*^–^ and the *lpa1* traits has been meanwhile obtained (Sparvoli et al., 2016). In our view, consumers will be highly interested in such innovative food formulations containing the *lec^–^ lpa1* flour trait. Future applications in drinks, fermented products, or spreading creams are among the targets. The presence of an active α-AI might very likely further contribute to even more healthy new bean-based products.

## Protein quality improvement

Protein quality of foods is assessed based on protein digestibility, bioavailability of amino acids, and amounts/proportions of essential amino acids (Gilani et al., 2012; Vaz Patto et al., 2015). Protein quality in common beans, like in other pulses, is suboptimal with a low content of sulfur amino acids (Pandurangan et al., 2015; Nosworthy et al., 2017). Therefore, common beans are often combined with cereals to provide a balanced protein source. Improvement of protein quality of common beans therefore represents, in our view, an important future task. In order to improve protein quality different approaches have been so far applied, including dehulling, soaking, boiling, roasting, autoclaving, micronization, microwave cooking, extrusion cooking, fermentation, as well as germination (Alonso et al., 2000; Mubarak, 2005; Khattab and Arntfield, 2009; Maphosa and Jideani, 2017; Shi et al., 2018). These methods improve protein digestibility by the inactivation of protease inhibitors, or lectins, or by protein denaturation thereby enhancing accessibility of susceptible sites to proteolysis (Linsberger-Martin et al., 2013; Drulyte and Orlien, 2019). In addition, screening for more digestible phaseolin (the most abundant common bean storage protein) isoforms has been also previously proposed as an alternative approach to improve protein quality (Montoya et al., 2008a,b,c, 2009). Although ultrafiltration does not inactivate ANFs, the technique is, however, able to remove and separate ANFs into different fractions (Avilés-Gaxiola et al., 2018). Considering, the feasibility from the technological and economic points of view, the soaking coupled to the heating of beans is an efficient way to increase protein digestibility and also desirable sensory properties (Khattab and Arntfield, 2009; Ferreira et al., 2014; Drulyte and Orlien, 2019). However, highly intensive processing deteriorates the nutritional quality of protein foods. This will reduce the essential amino acids bioavailability, inducing protein-protein interactions and cause amino acid racemization (Sá et al., 2022). In addition, it causes the loss of the potential gut protective effects of the inhibitors; in fact, undigested protein ends up in the colon and is fermented by gut microbiota and has a beneficial effect on human health (Kårlund et al., 2021).

A promising novel strategy to increase the content of sulfur amino acids in common bean seeds is also modifying protein fractions by decreasing particularly those fractions with a low content of limiting amino acids. Progressive deficiency in major seed proteins, phaseolin and lectins (*phsl*^–^ and *lec*^–^ traits), results in a significant increase in sulfur amino acid concentration, with cysteine concentration elevated by up to 70% and the methionine concentration by 10–20% (Taylor et al., 2008). These characteristics were further maintained when in a breeding program with a cultivated cultivar germplasm lines were generated using these deficient genotypes (Viscarra-Torrico et al., 2021). Breeding lines carrying the *phsl*^–^ and *lec*^–^ traits have been also recently investigated by Giuberti et al. (2019) for particularly increasing the zinc and iron content in beans. Seeds of twelve phytohemagglutinin-E-free bean lines carrying the mutations *low phytic acid*, phytohemagglutinin L-free, α-amylase inhibitors-free, phaseolin-free, and reduced amount of condensed tannins were introgressed and differently combined in seven genetic groups and analyzed for their nutrient composition. The study provided first evidence that the association of these genetic traits might indeed help in increasing iron and zinc seed content in bean biofortification.

## Characterization of *low phytic acid* bean genotypes

Characterization of common beans low in PA (*lpa* genotype) has recently attracted much attention. Specifically, stress-tolerance of *lpa* beans has become an interesting research focus (Punjabi et al., 2018; Raboy, 2020; Losa et al., 2022). Lowering the phytate content in plants can potentially cause phenotypic changes as well as a loss of the antioxidative function of PA during abiotic stress conditions. This important aspect should, in our view, not be overlooked when developing any new *lpa* bean lines (Colombo et al., 2022). In plant vacuoles, PA chelates excess iron, preventing harmful Fenton-type reactions (Doria et al., 2009). During this reaction, ferrous and/or ferric cations catalytically decompose hydrogen peroxide (H_2_O_2_) generating phytotoxic reactive oxygen radicals (ROS) (Das et al., 2015; Kunert and Foyer, 2022). Stable H_2_O_2_ can diffuse across cell membranes and vacuoles act as a H_2_O_2_ sink, with the uptake facilitated by tonoplast aquaporins (Smirnoff and Arnaud, 2019). However, more convincing evidence is, in our view, still needed to confirm this antioxidative role of PA, particularly under abiotic stress conditions. Findings so far supporting this role includes lowered drought sensitivity in high phytate containing bean lines (Hummel et al., 2018) and low-phytate (*lpa*) maize mutants having increased drought sensitivity (Badone et al., 2012). Additionally, maize mutants defective in the *ZmMRP4* (*Zea mays* Multidrug Resistance Protein 4) gene, coding for a PA transporter (Nagy et al., 2009), had a lower germination capacity coupled with higher levels of free radicals in the embryos (Shi et al., 2007; Doria et al., 2009). In contrast, higher drought tolerance in *lpa* mutants have also been reported (Nagy et al., 2009; Chiozzotto et al., 2018). A possible change in antioxidant transport into the vacuole in these mutants might be, in our view, an interesting future research target. In addition, *osipk1* (*Oryza sativa* inositol polyphosphate kinase 1) rice lines, mutated in the *IPK1* (inositol polyphosphate kinase 1) gene involved in the PA biosynthetic pathway had improved tolerance to both salt and drought stress while agronomic traits and seed viability were unaffected (Jiang et al., 2021). Studies in fish have also shown sensitivity of proteases to PA (Khan and Ghosh, 2013). We are currently also investigating if a low PA content activates proteolytic vacuolar processing enzymes involved in H_2_O_2_ degradation and programmed cell death (Hatsugai et al., 2015; Vorster et al., 2019; Yamada et al., 2020). Also, since findings of the antioxidative function is still unclear, we also think that testing in the future a larger number of these mutant genotypes is an urgent requirement to provide a better understanding of the response of *lpa* genotypes to stress conditions and the potential advantages from these genotypes, even if associated with moderate yield reductions (Raboy, 2020).

## *Low phytic acid* bean genotypes and pleiotropic effects

An increased iron bioavailability of common bean *lpa1* mutants has been successfully demonstrated in an *in vivo* study with human volunteers (Petry et al., 2013). Besides PA, polyphenols are, however, also a major constrain to iron bioavailability (Petry et al., 2010; Tako et al., 2014; Hart et al., 2017; Wiesinger et al., 2019), although not all polyphenols inhibit iron absorption (Hart et al., 2020). While supplying a diet with *lpa1* beans is, indeed, beneficial to iron absorption, *lpa1* beans also cause adverse gastrointestinal symptoms due to its hard-to-cook (HTC) phenotype concomitant with an increased thermal stability of lectins (Petry et al., 2016). The strength of the HTC defect in *lpa1* seeds depends, however, on how strong the effect the *lpa1* mutation has on the thermal stability of seed lectins and is further only problematic in genetic backgrounds harboring the PHA-L lectin alone, which is not very common. So far no significant effect on lectin thermal stability was found when the *lpa1* mutation is in a genetic background harboring both PHA-L and PHA-E, which is in most of bean genotypes or in a PHA-E background alone (Cominelli et al., 2020). These recent results are, indeed, important already helping to select superior traits in a breeding program aimed to nutritionally improve common beans. More recently, additional common bean *lpa* mutants, but less effective in PA reduction than *lpa1*, have been also isolated (Cominelli et al., 2018). They are currently tested for their cooking and nutritional properties in order to develop more useful biofortified beans devoid of negative traits. In our view, these isolated *lpa* bean crops have the great potential to benefit populations at risk of iron and zinc deficiency particularly evident in less developed countries. In contrast, bean crops with higher amounts of phytate will have health benefits in societies where more bioavailable forms of iron are present in the diet and with cancer and obesity a major health risk (Blair, 2013).

Furthermore, *lpa1* beans have also a lower zinc retention during boiling of soaked beans. This strongly suggests that zinc from the cotyledon possibly interacts with PA and prevents excessive zinc losses in the soaking and cooking water. In *lpa* beans, zinc might not interact much with the limited amounts of PA remaining which causes larger zinc losses in the soaking and cooking water (Hummel et al., 2020). In our view, deeper exploration of possible unexpected pleiotropic effects of the *lpa* mutation is urgently required by evaluating different types of *lpa* mutants and also to evaluate if effects are directly related to a PA decrease, or more related to the regulatory roles of components involved in PA biosynthetic pathway can play (Sparvoli and Cominelli, 2015; Freed et al., 2020).

## What next

For a transition from animal to plant proteins, more genetic resources are, in our view, urgently required by testing, for example, more mutant lines low in AFNs, including field testing. In this regard, identifying more mutants low in PA by possibly also exploring alternative ways to obtain such mutants should be among the targets. For example, mutations in genes belonging to the group 3 of the plant sulfate transporter family can also cause the *lpa* genotype (Sacchi and Nocito, 2019). Due to their beneficial effects at low concentrations, there is also a general agreement that ANFs in diets should not be reduced to zero levels. Highly important will be to establish threshold levels of each ANF that can be safely included in diets for specific population groups Geraldo et al. (2022). In healthy people eating a balanced diet, any PA effects on the iron, zinc, and manganese status is generally minimal not causing any nutrient deficiencies. However, the antioxidative role should thereby not be ignored. PA exhibits not only anti-cancer properties but also positively impacts cholesterol and blood sugar levels (Onomi et al., 2004; Nissar et al., 2017). Finally, we think that to work out a strategy with a genetic approach combining each single specific and nutritionally relevant trait may will be of outmost importance allowing to overcome many of the limiting factors commonly met when using common bean. Potentially it would mean, in our view, to develop common bean lines with the following characteristics: biofortified (*lpa*, *white seed coat–wsc-*), with increased protein quality (*phsl*^–^, *lec*^–^), not toxic (*lec*^–^), with potential hypoglycemic effects (αAI). Such material would be a valuable genetic reservoir of combinations to assess the true functional role of specific seed bioactive components directly in the food matrix.

## Data availability statement

The original contributions presented in this study are included in the article/supplementary material, further inquiries can be directed to the corresponding author.

## Author contributions

ECo and FS proposed and designed the review. ECo, FS, SL, and KK wrote the first draft of the manuscript. All authors contributed to the revision and the final version of the manuscript and approved the submitted version.

## Footnotes

1
https://www.fao.org/nutrition/%20nutrition-education/food-dietary-guidelines/en/
